# Clinical Outcomes of Peripheral Iridotomy in Patients with the Spectrum of Chronic Primary Angle Closure

**DOI:** 10.1155/2013/828972

**Published:** 2013-06-26

**Authors:** Ricardo J. Cumba, Kundandeep S. Nagi, Nicholas P. Bell, Lauren S. Blieden, Alice Z. Chuang, Kimberly A. Mankiewicz, Robert M. Feldman

**Affiliations:** ^1^Ruiz Department of Ophthalmology and Visual Science, The University of Texas Medical School at Houston, 6431 Fannin Street, MSB 7.024, Houston, TX 77030, USA; ^2^Ophthalmology Department, Medical School, University of Puerto Rico, Medical Science Campus, P.O. Box 365067, San Juan, PR 00936, USA; ^3^Department of Ophthalmology, The University of Texas Health Science Center at San Antonio, 7703 Floyd Curl Drive, Mail Code 6230, San Antonio, TX 78229, USA; ^4^Robert Cizik Eye Clinic, 6400 Fannin Street, Suite 1800, Houston, TX 77030, USA

## Abstract

*Purpose*. To evaluate outcomes of peripheral iridotomy (PI) for initial management of primary angle closure suspects (PACS), chronic primary angle closure (CPAC), and chronic primary angle closure glaucoma (CPACG). *Patients and Methods*. Seventy-nine eyes with PACS, CPAC, or CPACG and better than 20/50 visual acuity that underwent PI as initial management were included. Eyes with previous acute angle closure attacks, laser trabeculoplasties, surgeries, or intraocular injections were excluded. Additional treatments, glaucomatous progression, intraocular pressure, visual acuity, and the number of medications were evaluated. *Results*. The mean followup was 57.1 ± 29.0 months (range 13.8–150.6 months). Sixty-eight eyes (86.1%) underwent additional medical, laser, or surgical treatment. Forty eyes (50.6%) underwent lens extraction due to reduced visual acuity. The mean 10× logMAR visual acuity score for all patients significantly declined from 0.94 ± 1.12 at baseline to 1.83 ± 3.49 (*N* = 79, *P* = 0.0261) at the last followup. *Conclusions*. Most patients who undergo PI for CPAC spectrum will require additional intervention for either IOP lowering or improvement of visual acuity. This suggests that a procedure that not only deepens the angle but also lowers IOP and improves visual acuity would be desirable as further intervention could be avoided. Evaluation of techniques that achieve all 3 goals is warranted.

## 1. Introduction

Primary angle closure glaucoma (PACG) is a leading cause of bilateral blindness worldwide [1]. The disease is estimated to affect 16 million people, with 4 million bilaterally blind [2]. The chronic primary angle closure (CPAC) spectrum of disease ranges from primary angle closure suspects (PACS) to CPAC to chronic primary angle closure glaucoma (CPACG).

PACS consist of eyes with anatomically narrow angles potentially predisposing to angle closure. Once closure has developed (as evidenced by elevated intraocular pressure (IOP), peripheral anterior synechia (PAS), trabecular pigment smudging, or other signs of true apposition of iris to trabecular meshwork),CPAC has occurred. Chronic primary angle closure glaucoma (CPACG) is diagnosed when, in addition to CPAC, glaucomatous optic neuropathy is present, as evidenced by visual field, nerve fiber layer, or optic nerve damage [3].

Treatment of the CPAC spectrum is directed toward 2 goals: (1) eliminate the mechanism of angle closure and (2) control any remaining IOP elevation. Peripheral iridotomy (PI) is currently the first line of treatment [4]. If PI does not improve angle anatomy, iridoplasty may be performed to open the angle, but this too may not be a permanent solution [4–6]. Even with correction of the anatomy, trabecular function may not be fully restored due to persisting damage from chronic trabecular contact with the iris. Residual elevated IOP is typically treated medically with aqueous suppressants and/or uveoscleral outflow enhancers. If medications fail to reduce IOP to the desired target, more aggressive surgical intervention may be indicated, typically glaucoma filtering surgery.

It has been suggested that lens extraction (LE) may be effective as an initial treatment for the CPAC spectrum [7–9]. The replacement of the crystalline lens with a thinner prosthetic intraocular lens (IOL) creates additional space behind the iris, reducing lens-iris contact and the resultant relative pupillary block. However, incisional intraocular surgery carries significant risk, making clear lens extraction for CPAC/CPACG controversial. On the other hand, laser PI and/or chronic topical ophthalmic medication use may accelerate lens opacification [10–13], resulting in progressive vision loss and need for further intervention. Therefore, it would be important to know the frequency of LE after iridotomy to help weigh the risks and benefits of each treatment option in the management of the CPAC spectrum. 

The aim of the current study is to evaluate the outcomes of PI for the initial management of the CPAC spectrum in phakic patients with good vision at the time of iridotomy.

## 2. Patients and Methods

This retrospective case series was conducted at the Robert Cizik Eye Clinic of the Ruiz Department of Ophthalmology and Visual Science at The University of Texas Medical School at Houston, Houston, USA. The Institutional Review Board (The University of Texas Health Science Center Committee for the Protection of Human Subjects) determined that this study was exempt from review prior to chart search and review. All research adhered to the tenets of the Declaration of Helsinki and was HIPAA compliant.

A computerized search of the practice database was performed to identify all patients who had PI from January 1995 to December 2005 for the CPAC spectrum of disease. Eyes with best corrected visual acuity better than 20/50 and PI were identified. Eyes with less than 1 year of followup, a history of acute angle closure, and previous laser trabeculoplasty, intraocular incisional surgery, extraocular surgery, or intraocular injections were excluded.

The CPAC spectrum was divided into 3 groups (as adapted from Foster et al. [3]):

 PACS: a narrow, potentially occludable angle with no identifiable anatomic or syndrome-related causes other than pathological angle closure;
 CPAC: PACS with the presence of peripheral anterior synechiae (PAS), IOP ≥ 21 mmHg, or on IOP-lowering medications;
 CPACG: CPAC with glaucomatous visual field, nerve fiber layer, or optic disc damage.

### 2.1. Treatment

All eyes diagnosed in the CPAC spectrum were initially treated with Nd:YAG laser PI [14] by 2 of the authors who are fellowship-trained glaucoma specialists (RMF, NPB). Repeated laser or incisional PI was typically performed when the initial PI was found to be nonpatent. Iridoplasty was performed [15] when the angle did not deepen after PI on dark room gonioscopy. IOP-lowering medications were discontinued by the 1-month followup for all eyes. Pilocarpine was not used as a medical therapy.

### 2.2. Data

Demographics and baseline ocular characteristics, including Snellen best corrected visual acuity (BCVA), IOP (applanation), cataract grade (1–4+, based on color), number of IOP-lowering medications, Spaeth gonioscopic angle classification [16] performed in a dark room with and without indentation, and diagnosis at presentation were recorded. During the followup office visits, Snellen BCVA, cataract grade, IOP, number of IOP-lowering medications, and clinical impression of visual field/optic disc progression were recorded. Snellen BCVA was performed in a dark room by typical clinical protocol. Optic discs were analyzed by comparing examination to baseline simultaneous stereo optic disc photographs. Progression to glaucoma was determined clinically using Hodapp-Parrish-Anderson criteria [17]. During the study period, the clinic transitioned from Humphrey 24-2 Full Threshold to Humphrey 24-2 SITA-Standard (Carl Zeiss Meditec, Inc., Dublin, CA, USA). The switch in visual field testing strategies during the study period limits the ability to perform formal visual field change analyses. Followup gonioscopy was typically performed during the first post-PI month, and additional treatment for angle deepening (iridoplasty) was undertaken if the angle was still considered occludable. The closest visits correlating to postoperative times of 6 months, 12 months, and one year thereafter were used as the study visits. Any interval ocular surgery, including date and type of surgery (i.e., iridoplasty, trabeculectomy, laser trabeculoplasty, cataract extraction, combined cataract extraction and trabeculectomy), was recorded. 

### 2.3. Outcomes

The primary outcome variables were glaucomatous visual field/optic disc progression and the need for additional treatments. Additional treatments were further classified into the following categories: (1) medical IOP-lowering therapy; (2) additional glaucoma surgery; or (3) lens extraction surgery. Secondary outcome variables included change in angle status, IOP, BCVA, change in cataract grading, and a number of IOP-lowering medications from baseline to the last visit. Additionally, if cataract extraction or glaucoma surgery was performed, the visit prior was taken as the last visit data.

### 2.4. Statistical Analysis

Demographic and baseline clinical data were summarized by mean (± standard deviation) or frequency (percentage). The primary outcome variables were reported as frequency (percentage). The time to additional treatment was defined as the number of months following initial laser PI until the introduction of any treatment (medication, glaucoma surgery, or lens extraction). Similarly, time to additional glaucoma treatment was defined as the number of months after initial laser PI until the introduction of IOP-lowering medication or glaucoma surgery was performed, and time to lens extraction was defined as the number of months after initial laser PI until cataract surgery. Time to progression was defined as the number of months after PI until visual field and/or optic disc progression was observed. Snellen BCVA was converted to the 10× logMAR scale, −log⁡(BCVA) × 10. Count fingers was coded as 20/1500, hand motion as 20/4000, and light perception as 20/8000. The paired *t*-test was performed to assess pre- to post-PI means. The Student's two-sample *t*-test was used for other comparisons. Logistic regression analysis was used to examine the effect of using IOP-lowering medication prior to PI on the requirement of IOP-lowering medications after PI.

All statistical tests were conducted at a 5% level of significance. Analysis was performed using SAS software version 9.2 for Windows (SAS Institute, Inc., Cary, NC, USA).

## 3. Results 

### 3.1. Baseline Characteristics

A total of 79 eyes of 52 patients with PACS (25 eyes), CPAC (30 eyes), or CPACG (24 eyes) met inclusion/exclusion criteria. There were 34 women (65.4%), and the mean age of the patients was 64.6 ± 12.5 years. The study included 22 White (42.3%), 15 Black (28.9%), 11 Hispanic (21.2%), and 4 Asian (7.7%) patients. The baseline ocular characteristics are shown in Table 1.

Forty of the total 79 eyes were right eyes (50.6%). All eyes (by eligibility criteria) had BCVA better than 20/50, while 55 eyes (69.6%) were 20/25 or better at the baseline. Seventy of the 79 eyes had cataract grading greater than 0 at baseline. Of these, 49 were classified as CPAC or CPACG. Higher graded nuclear sclerosis was more likely to be found in eyes with more advanced degrees of angle closure (PACS versus CPAC/CPACG, *P* = 0.0205). 

Thirty-six eyes were on IOP-lowering therapy at the time of PI. The use of IOP-lowering medications prior to PI was correlated with disease severity; that is, no PACS patients were on IOP-lowering medications before PI, while 18 (60%) CPAC and 18 (75%) CPACG patients were on IOP-lowering medications before PI (*P* < 0.0001). Fifteen medically untreated eyes had an IOP ≥ 21 mm Hg. The mean IOP (for all eyes, with and without IOP-lowering treatment at baseline) was 19.6 ± 5.5 mm Hg, and the mean number of IOP-lowering medications was 0.75 ± 0.98.

All eyes included in the study had a 20-degree or narrower angle by Zeiss gonioscopy, with the exception of 2 CPAC eyes. Of these, one eye had an IOP of 23 mm Hg with PAS, and the other eye had an IOP of 26 mm Hg without PAS. Both of these eyes had plateau-type appearance. Ten eyes (5 CPAC and 5 CPACG) had some PAS.

### 3.2. Treatment

All eyes had a laser PI as the initial management. Three eyes (4%; 3 CPAC) required repeated PI due to nonpatency. In one eye, repeated PI was performed at 4 months and the remaining 2 eyes had repeated PI at the 1-year followup visit. Twelve of the 79 study eyes (15.2%; 3 PACS, 5 CPAC, and 4 CPACG) had iridoplasty after PI.

### 3.3. Outcomes

The mean followup time was 57.1 ± 29.0 months, with a range of 13.8–150.6 months. The mean number of followup visits was 4.2 ± 3.2 per year (2.8 ± 1.7 visits for the PACS group, 4.0 ± 1.8 for CPAC, and 6.3 ± 4.8 for CPACG) with a median of 3.6 per year. Sixty-eight of the 79 eyes (86.1%) required additional medical, laser, or surgical treatment. A breakdown of the additional treatments by diagnostic group is presented in Table 2. There were no other ocular surgical interventions in the study population other than cataract extraction or filtration surgery.

#### 3.3.1. Medical Therapy

One month after initial PI, 50 eyes (63.3%) required at least 1 IOP-lowering medication. The mean duration from initial PI to introduction of medication was 9.7 ± 12.7 months (*N* = 50). The mean number of medications required at the last followup (1.03 ± 1.11) was significantly increased from baseline (0.75 ± 0.98; *P* = 0.0194). At the last followup, 25 (31.6%) eyes were on more IOP-lowering medications than baseline. Additional details on medical therapy can be seen in Table 3. Eyes that were on medications before PI were 5.8 times more likely to be on medications after PI (*P* = 0.0012; 95% CI, 2.0–16.6). 

#### 3.3.2. Surgical Therapy

Forty-eight eyes (60.8%; 13 PACS, 18 CPAC, and 17 CPACG) underwent additional surgical interventions after initial PI. In most cases, additional surgical intervention occurred later than 1 month after PI. However, 2 eyes had additional glaucoma surgery within 1 month of initial PI (was not included as additional medical therapy). One eye had an IOP spike (48 mm Hg) immediately after the initial PI that could not be controlled medically, and trabeculectomy was performed urgently. IOP was controlled without additional glaucoma treatment throughout the next 7 years of followup. The other eye, which was on 4 IOP-lowering medications prior to PI, had elevated IOP (31 mm Hg) at 3 weeks after PI. A laser trabeculoplasty was performed. This eye was continuously treated with 4 IOP-lowering medications during 6 years of followup, with no glaucomatous progression observed. All 13 PACS eyes that had additional surgery had cataract extraction alone and no glaucoma surgery; 9 CPAC and 3 CPACG eyes had cataract extraction alone; 2 CPAC and 6 CPACG eyes had glaucoma surgery alone; 5 eyes had cataract extraction and glaucoma surgery combined; 7 eyes had glaucoma surgery followed by cataract extraction; and 3 eyes required glaucoma surgery after cataract extraction. See Table 2 for a breakdown. 

#### 3.3.3. Cataract Extraction

Forty eyes (50.6%) underwent cataract extraction for an indication of reduced visual acuity believed secondary to cataract. In the eyes that underwent cataract extraction, BCVA (10× logMAR) decreased from 1.25 ± 1.15 at baseline to 3.15 ± 3.72 (*P* = 0.0017) immediately prior to cataract extraction. Visual acuity improved to 2.13 ± 3.27 (*P* = 0.1084) after cataract extraction. One eye developed bullous keratopathy with resultant visual acuity of hand motion. After excluding this eye, the mean BCVA at the last followup was 1.69 ± 1.81 (*N* = 39, *P* = 0.1666), which was not statistically different from the baseline. The mean duration from initial PI to cataract extraction was 31.7 ± 22.4 months (*N* = 40).

#### 3.3.4. Glaucomatous Progression

Fourteen eyes (17.7%) had glaucomatous progression during the course of followup; 4 eyes demonstrated visual field progression only, 6 eyes optic disc progression only, and 4 both (Table 2). The mean duration from initial PI to progression was 28.9 ± 18.3 months. Two eyes were PACS eyes (8.0% of 25 PACS eyes), 3 were CPAC eyes (10% of 30 CPAC eyes), and 9 were CPACG eyes (38% of 24 CPACG eyes).

#### 3.3.5. Effect of Iridoplasty

Table 2 also presents the outcomes for eyes with PI alone (67 eyes) and those with PI followed by iridoplasty (12 eyes). There were no statistically significant differences between these 2 groups.

#### 3.3.6. Gonioscopy Examination

In addition to baseline, 69 eyes (87.3%) had gonioscopy examinations by the 1-month followup visits, while the remaining 10 eyes were examined later. In 53 eyes (67.2%), the angle deepened by at least 10 degrees after PI (Table 3). However, there was neither a relationship between the amount of deepening after PI and the need for IOP-lowering medications (*P* = 0.8270, Table 4), nor any relationship with change in number of medications from baseline (*P* = 0.5019, Table 4).

#### 3.3.7. IOP and Number of IOP-Lowering Medications

IOP and number of IOP-lowering medications at each time point are summarized in Table 5. Six months after PI, the mean number of IOP-lowering medications for all eyes was not statistically different than baseline. Although mean IOP was reduced by 1.9 (±6.8) mm Hg at the last visit, 25 eyes (31.6%) were on more medications at the last visit than at baseline, while 11 eyes (13.9%) were on less medications (Table 3).

#### 3.3.8. Best Corrected Visual Acuity

BCVA in 10× logMAR scale was 0.94 ± 1.12 at baseline for all eyes (*N* = 79). At the last followup, 23 (29.5%) eyes lost 2 or more lines of vision while 50 (63.3%) eyes had an increase in cataract grade (Table 3). The mean visual acuity for all patients significantly declined to 1.83 ± 3.49 (*N* = 79,  *P* = 0.0261) at the last followup (mean followup duration 57.1 ± 29.0 months). Two eyes had BCVA worse than 16 (20/800 equivalent) at the last followup: 1 due to bullous keratopathy (as mentioned above) and the other due to a cerebrovascular accident. If these eyes are excluded, the mean BCVA was 1.34 ± 1.59, which is still statistically significantly worse than baseline (*N* = 77, *P* = 0.0370).

## 4. Discussion

Peripheral iridotomy (PI) is currently the initial treatment for cases of CPAC. If after initial PI IOP remains elevated, patients are usually treated with glaucoma medications first and then surgery as necessary [4]. However, there is not enough data on the long-term outcomes after initial PI to determine if the current treatment algorithm is effective in preventing vision loss in patients who present with good visual acuity.

This case series demonstrates that PI alone does not prevent patients from requiring additional treatment or surgery. Eyes that were on medications before PI were 5.8 times more likely to be on medications after PI. Six months after PI, the number of medications used before surgical treatment was not any different from baseline. Of the 79 study eyes, 68 (86.1%) required additional medical and/or surgical intervention, including IOP-lowering medications, trabeculectomy, or cataract extraction. Twenty-four eyes with CPAC (80.0%) and 20 eyes with CPACG (83.3%) required additional glaucoma treatment after initial PI. Only 7 (28.0%) PACS eyes required additional glaucoma treatment but 13 (52.0%) required cataract extraction. PI alone generally failed to control IOP. By the 6-month followup visit, the mean number of IOP-lowering medications was not reduced from preiridotomy levels. Additionally, 23 CPAC/CPACG eyes (29.1%) required filtering surgery. 

In the current study, 51 of 79 eyes (64.6%) required additional medical and/or surgical treatment to control IOP (including 83.3% (20 of 24 eyes) in the CPACG group). Despite these interventions, 2 PACS eyes (8% of 25 PACS eyes), 3 CPAC eyes (10% of 30 CPAC eyes), and 9 CPACG eyes (38% of 24 CPACG eyes) demonstrated glaucomatous progression. The results of this study regarding the need for additional intervention for IOP lowering are similar to the results of Rosman et al., a North American population [18]. In their study, all 80 eyes (100%) required medical and/or surgical intervention to control IOP, despite a patent PI. Thirty-three (41.3%) only required additional medication, 22 (27.5%) required additional laser surgical intervention, and 25 (31.3%) required additional incisional surgical intervention [18]. This discrepancy may be explained by the fact that Rosman's study included only subjects with glaucoma and did not include PACS or CPAC eyes. 

In the current study, 7 of 25 PACS eyes (28.0%), 24 of 30 CPAC eyes (80.0%), and 20 of 24 CPACG eyes (83.3%) required additional medical and surgical glaucoma treatments; 0 of 25 PACS eyes (0.0%), 9 of 30 CPAC eyes (30%), and 14 of 24 CPACG eyes (58.3%) required additional glaucoma surgery. In another study by Peng et al. analyzing a spectrum of Vietnamese patients with CPAC, 7.1% (17 of 239 eyes) with PACS, 42.4% (42 of 99 eyes) with CPAC, and 100% (21 of 21 eyes) with PACG required medical and/or surgical intervention after PI. Of these eyes, 0.4% (1 eye) with PACS, 8.1% (8 eyes) with CPAC, and 42.9% (9 eyes) with CPACG underwent filtering surgery [19]. These trends in results are similar between the 2 studies, except that the percentages of eyes requiring further treatment and the percentage of eyes undergoing filtering surgery in the CPAC and CPACG groups are higher in the current study, probably because Peng et al. did not consider cataract extraction as a treatment for narrow angles.

The PACS group in both the current study and Peng et al.'s did much better in terms of IOP control after PI. In the current study, 7 eyes (28.0%) developed ocular hypertension, requiring additional medical therapy with 2 eyes progressing to glaucomatous damage (of 25 PACS eyes; 8.0%). In the study by Peng et al., 9 of 239 PACS (3.8%) eyes progressed. The combined results of these 2 studies indicate that continued vigilance is warranted for PACS patients treated with PI. Although PACS eyes are not likely to require surgical intervention for glaucoma, they are likely to develop visually significant cataract requiring extraction. Thirteen of the 25 PACS (52%) eyes underwent cataract extraction in the current study. Additionally, Peng et al. included a risk factor analysis for progression of disease and found that lens extraction was potentially protective of progression to PAC from PACS [19].

Gonioscopy examination revealed that angle deepening had no effect on the need for IOP-lowering medications. Fifty-three eyes (67.2%) showed that the angle was deepened by at least 10 degrees after PI. However, the degrees of angle deepening after PI neither affected the need for IOP-lowering medications nor change in number of medications from baseline. Treatment with iridoplasty also did not result in any changes in outcomes. However, the number of patients with iridoplasty in this study was small (*N* = 12). 

While trabeculectomy (after failure of medical therapy) is traditionally used to treat CPACG, lens extraction (with or without goniosynechialysis) has been proposed as an alternative. It is known that the lens is integral in the pathogenesis of CPAC [20], and in some cases lens extraction alone may be adequate to control CPACG [7–9]. In some cases, combined surgery may be advantageous for the control of IOP, deepening of the angle, and visual improvement. Alsagoff et al. reported that of 44 eyes with angle closure that required surgical treatment, 20 (45.5%) had cataract extraction as part of a combined filtering surgery [21]. Of the CPAC eyes in the current study, 16 (53.3%) underwent cataract extraction: 9 had cataract extraction alone, 3 had combined glaucoma/cataract extraction surgery, 3 had glaucoma surgery followed by cataract extraction, and only 1 had cataract extraction followed by glaucoma surgery. Of the CPACG eyes, 11 (45.8%) underwent cataract extraction: 3 had cataract extraction alone, 2 had combined glaucoma/cataract extraction surgery, 4 had glaucoma surgery followed by cataract extraction, and 2 had cataract extraction followed by glaucoma surgery. However, all 13 (52.0%) PACS eyes that underwent surgical intervention had cataract extraction alone, without the need for combined or later glaucoma procedures. These findings confirm those of Peng et al. in that early lens extraction may potentially be a viable preventative treatment of CPAC [19].

Not only did PI fail to control IOP without medications and did not prevent the need for further glaucoma treatment, but visual acuity loss continued uninterrupted, as evidenced by more than half of the eyes (50.6%; 40 of 79 eyes) requiring cataract extraction in the course of the study. This rate of cataract extraction is probably higher than expected, given the good initial visual acuity required for inclusion in the study. However, there exists a potential for bias because the surgeon was aware of the angle closure diagnosis, which may have lowered the threshold for cataract extraction. In other words, lens extraction could have been performed at a lower threshold of visual acuity loss in this study population than in a nonangle closure population. 

At baseline, visual acuity (10× logMAR) was 0.94 ± 1.12, and even though 40 eyes underwent cataract extraction, BCVA at last followup visit declined to 1.83 ± 3.49, mostly related to 2 eyes with severe visual loss. There was no significant difference in BCVA between those who underwent cataract extraction and those who did not. Of the eyes with severe visual loss, 1 had a trabeculectomy complicated by hypotony with a flat anterior chamber and choroidal effusions. The patient later underwent cataract extraction and developed pseudophakic bullous keratopathy; the cornea never recovered, and the patient refused corneal transplantation. The second eye, which did not have cataract extraction, developed poor visual acuity after the patient suffered from a cerebrovascular accident. If these 2 eyes are excluded, visual acuity still declined but not to a clinically significant degree. Significant loss of acuity is uncommon after cataract extraction. It is unknown if eyes with CPAC spectrum disease are at higher risk than the general population for complications which may result in loss of acuity.

This study differs from previous studies in that the study population included only eyes with good visual acuity and evaluated patient outcomes in terms of additional surgery, including cataract extraction, intraocular pressure control, and glaucoma progression. In clinical practice, the visual acuity loss from poorly controlled angle closure and the visual loss from cataracts often develop concurrently. Additionally, this study does not include eyes with a history of previous acute angle closure attacks. Inclusion of eyes with previous acute angle closure attacks combines residual angle closure and chronic angle closure occurring *de novo,* which may have different clinical implications. 

The current study has several limitations. As is typical in retrospective studies, followup periods and data available varied. The loss of followup is an inherent characteristic of retrospective studies. Eyes with less than a year of followup were excluded, which could bias the results because patients who did well may have returned to referring physicians for care, while those who were not doing well either remained in the practice or were referred back for further care. Also, since the CPAC spectrum diseases are generally asymptomatic, patients may have been treated with PI and lost to followup care entirely, which may bias the results in an unknown way. The vast majority of patients in the study were followed continuously and came from internal referrals. 

Because both eyes of a patient were included if eligible in this study, this may have biased the estimation of standard deviations of outcome variables, as there was a correlation between eyes (*κ* = 0.6 for requiring additional treatment intervention). However, by including an additional 27 eyes, the estimation of the means gained some precision.

We were unable to adequately assess progression from PACS to CPAC based on the retrospective data available in the charts. The criteria of Foster et al. [3] could not be applied to the clinical situation due to the various presentations of the CPAC spectrum of disorders, as suggested by Sihota [22]. Thus, we are limited to discussing patient progression from PACS to CPACG, and statistical comparison of the 2 groups was beyond the scope of this paper. Because serial optic disc photos were not obtained, optic disc progression could not be later confirmed by reevaluating photographs because only a baseline set of photographs exists, except in cases where change was determined clinically. Similarly, although automated visual fields were used to determine progression, confirmation fields were rarely obtained. 

This study does not examine the direct progression of the anatomy of angle closure but rather analyzes surrogate measures of further clinical intervention, which ultimately will affect the patient. Gonioscopy is a very subjective technique, where intraobserver and interobserver variability is poor [23–25]. Additionally, this study is limited in its ability to evaluate the effect of PI on cataract progression as there is no appropriate control group.

Also, only 2 ophthalmologists were involved in the care and treatment of the patients, making generalizability of the results limited. Nevertheless, this study is unique in that it is the first study to provide information regarding clinical outcomes after initial PI in the entire CPAC spectrum of patients.

In conclusion, most patients who undergo PI for CPAC spectrum will require additional intervention for either IOP lowering or improvement of visual acuity. This suggests that a procedure that not only deepens the angle but also lowers IOP and improves visual acuity would be desirable in the treatment of PAC as further intervention could be avoided. Evaluation of techniques that achieve all 3 goals is warranted. 

## Figures and Tables

**Table 1:** Baseline ocular characteristics.

Variables	All eyes (*N* = 79)	CPAC spectrum disease		
		PACS (*N* = 25)	CPAC (*N* = 30)	CPACG (*N* = 24)
Best corrected visual acuity				

10× logMAR, mean ± SD	0.9 ± 1.1	0.7 ± 1.1	1.2 ± 1.1	0.9 ± 1.2
20/25 or better, *N* (%)	55 (69.7)	19 (76.0)	18 (60.0)	18 (75.0)

Cataract grade				

0, *N* (%)	9 (11.4)	4 (16.0)	5 (16.7)	0 (0.0)
1, *N* (%)	39 (49.4)	16 (64.0)	10 (33.3)	13 (54.2)
2, *N* (%)	21 (26.6)	4 (16.0)	10 (33.3)	7 (29.2)
3, *N* (%)	10 (12.7)	1 (4.0)	5 (16.7)	4 (16.7)

IOP				

IOP with or without medical treatment (mm Hg), mean ± SD	20.0 ± 6.0	16.0 ± 3.0	22.0 ± 5.7	21.6 ± 6.7
Number of IOP-lowering medications				
0, (*N*, %)	43 (54.4)	25 (100.0)	12 (40.0)	6 (25.0)
1, (*N*, %)	20 (25.3)	0 (0.0)	13 (43.3)	7 (29.2)
2, (*N*, %)	9 (11.4)	0 (0.0)	5 (16.7)	4 (16.7)
3, (*N*, %)	7 (8.9)	0 (0.0)	0 (0.0)	7 (29.2)
Mean ± SD	0.75 ± 0.98	0.0 ± 0.0	0.8 ± 0.7	1.5 ± 1.2

Gonioscopy examination				

Degrees of angle depth				
0, *N* (%)	9 (11.4)	0 (0.0)	5 (16.7)	4 (16.7)
10, *N* (%)	18 (22.8)	8 (32.0)	5 (16.7)	6 (25.0)
20, *N* (%)	50 (63.3)	17 (68.0)	18 (60.0)	14 (58.2)
30, *N* (%)	2 (2.5)	0 (0.0)	2 (6.7)	0 (0.0)
PAS, *N* (%)	10 (12.7)	0 (0.0)	5 (16.7)	5 (20.8)

PAS: peripheral anterior synechiae; IOP: intraocular pressure; CPAC: chronic primary angle closure; PACS: primary angle closure suspect; CPACG: chronic primary angle closure glaucoma.

**Table 2:** Summary of primary outcomes after PI.

Variable	All Eyes (*N* = 79)	PAC spectrum disease			Treatment	
		PACS (*N* = 25)	CPAC (*N* = 30)	CPACG (*N* = 24)	PI only (*N* = 67)	PI + Iridoplasty (*N* = 12)
Followup duration (months), mean ± SD	57.1 ± 29.0	54.4 ± 36.9	60.9 ± 26.3	55.1 ± 23.1	54.5 ± 36.3	71.7 ± 39.4
Additional medical and surgical treatments, *N* (%)	68 (86.1)	18 (72.0)	28 (93.3)	22 (91.7)	56 (83.6)	12 (100)
Additional glaucoma treatments, *N* (%)	51 (64.6)	7 (28.0)	24 (80.0)	20 (83.3)	42 (62.7)	9 (75.0)
Medical treatment only	28	7	15	6	24	4
Glaucoma surgery (GS)*	23	0	9	14	18	5
Cataract extraction (CE) procedure, *N* (%)	40 (50.6)	13 (52.0)	16 (53.3)	11 (45.8)	34 (50.8)	6 (50.0)
Additional surgery (CE or GS), *N* (%)	48 (60.8)	13 (52.0)	18 (60.0)	17 (70.8)	40 (59.7)	8 (66.7)
CE only	25	13	9	3	22	3
GS only	8	0	2	6	6	2
Combined CE and GS	5	0	3	2	5	0
GS first then CE	7	0	3	4	5	2
CE first then GS	3	0	1	2	2	1
Clinical impression of visual field/optic disc progression, *N* (%)	14 (17.7)	2 (8.0)	3 (10.0)	9 (37.5)	13 (19.4)	1 (8.3)
Visual field only	4	0	1	3	4	0
Optic disc only	6	1	1	4	5	1
Both visual field and optic disc	4	1	1	2	4	0

CE: cataract extraction; GS: glaucoma surgery; PACS: primary angle closure suspect; CPAC: chronic primary angle closure; CPACG: chronic primary angle closure glaucoma.

*Glaucoma surgery was performed after medical treatment, except for one PAC eye which was never on medications during followup.

**Table 3:** Summary of secondary outcomes.

Outcome variable	All Eyes (*N* = 79)	PACS (*N* = 25)	CPAC (*N* = 30)	CPACG (*N* = 24)
Gonioscopy examination after PI, *N* (%)				

Angle deepened by				
0 degrees	26 (32.9)	8 (32.0)	8 (26.7)	10 (41.7)
10 degrees	39 (49.4)	13 (52.0)	16 (53.3)	10 (41.7)
20+ degrees	14 (17.8)	4 (16.7)	6 (20.0)	4 (16.7)
Number of eyes with gonioscopy examination before or at 1-month visit	69 (87.3)	5 (20.0)	4 (13.3)	1 (4.2)

IOP change from baseline to last visit*				

Time between baseline and last IOP measurement (months), mean ± SD	34.1 ± 24.6	39.5 ± 19.4	38.1 ± 28.3	23.4 ± 22.2
IOP change (mm Hg), mean ± SD	−1.9 ± 6.8	0 ± 4.0	−2.8 ± 7.2	−2.6 ± 8.3
Number of eyes with change in IOP-lowering medications, *N* (%)				
Less medications	11 (13.9)	0 (0.0)	5 (16.7)	6 (25.0)
No change	43 (54.4)	19 (76.0)	14 (46.7)	10 (41.7)
More medications	25 (31.6)	6 (24.0)	8 (36.7)	8 (33.3)

Cataract grade change from baseline to last visit^¥^, *N* (%)				

No change	29 (36.7)	8 (32.0)	12 (40.0)	9 (37.5)
Increased by 1	38 (48.1)	14 (56.0)	15 (50.0)	9 (37.5)
Increased by 2	12 (15.2)	3 (12.0)	3 (10.0)	6 (25.0)

BCVA change from baseline to last visit^¥^				

Time between baseline and last BCVA measurement (months), mean ± SD Number of eyes with BCVA Change, *N* (%)^#^	41.4 ± 26.9	39.5 ± 19.4	43.4 ± 31.3	40.9 ± 28.7
Loss of 2 or more lines	23 (29.5)	7 (29.2)	7 (23.3)	9 (37.5)
Change within 2 lines	52 (66.7)	16 (66.7)	22 (73.3)	14 (58.3)
Gain 2 or more lines	3 (3.8)	1 (4.2)	1 (3.3)	1 (4.2)

LE: lens extraction; GS: glaucoma surgery; PACS: primary angle closure suspect; CPAC: chronic primary angle closure; CPACG: chronic primary angle closure glaucoma; BCVA: best corrected visual acuity.

*Last followup visit or the last visit before LE or GS.

^¥^Last followup visit or last visit before LE.

^#^Missing one PACS eye.

**Table 4:** Number of eyes requiring IOP-lowering medication and number of eyes with changes in IOP-lowering medication(s) by angle deepening after PI.

	Angle deepening					
	0 degrees (*N* = 26)	10 degrees (*N* = 39)	20+ degrees (*N* = 14)
Eyes requiring IOP-lowering medication(s)			
No, *N* (%)	9 (34.6)	15 (38.5)	5 (35.7)
Yes, *N* (%)	17 (65.4)	24 (61.5)	9 (64.3)
Change in number of IOP-lowering medication(s)			
Reduced, *N* (%)	4 (15.4)	5 (12.8)	2 (14.3)
Same, *N* (%)	16 (61.5)	22 (56.4)	5 (35.7)
Increased, *N* (%)	6 (23.1)	12 (30.8)	7 (50.0)

**Table 5:** Mean and standard deviation of IOP and number of medications at the scheduled followup visits^‡^.

Visit	IOP			Number of medications		
	PACS	CPAC	CPACG	PACS	CPAC	CPACG
Pre-PI	16.0 ± 3.0 (*N* = 25)	22.0 ± 5.7 (*N* = 30)	21.6 ± 6.7 (*N* = 24)	0.0 ± 0.0 1.0 (*N* = 25)	0.8 ± 0.7 (*N* = 30)	1.5 ± 1.2 (*N* = 24)
6 months	16.0 ± 3.4 (*N* = 16)	18.6 ± 4.7* (*N* = 24)	18.7 ± 7.4 (*N* = 10)	0.06 ± 0.25 (*N* = 16)	0.68 ± 0.80 (*N* = 25)	0.80 ± 0.63 (*N* = 10)
12 months	15.3 ± 3.9 (*N* = 18)	19.0 ± 5.7* (*N* = 22)	17.7 ± 6.3 (*N* = 11)	0.11 ± 0.32 (*N* = 18)	0.77 ± 0.92 (*N* = 22)	1.18 ± 1.17 (*N* = 11)
24 months	16.9 ± 5.5 (*N* = 15)	19.0 ± 4.0* (*N* = 19)	16.9 ± 5.6 (*N* = 10)	0.07 ± 0.26 (*N* = 15)	0.74 ± 0.73 (*N* = 19)	1.70 ± 1.16 (*N* = 10)
36 months	15.3 ± 2.4 (*N* = 12)	16.4 ± 3.1* (*N* = 10)	19.8 ± 5.7 (*N* = 8)	0.23 ± 0.44 (*N* = 13)	0.90 ± 0.74 (*N* = 10)	1.50 ± 1.60 (*N* = 8)
48 months	15.2 ± 3.7 (*N* = 10)	19.4 ± 3.9 (*N* = 9)	15.8 ± 3.6 (*N* = 5)	0.00 ± 0.00 (*N* = 10)	1.00 ± 0.82 (*N* = 10)	1.80 ± 1.10 (*N* = 5)
60 months	14.0 ± 6.1 (*N* = 4)	18.3 ± 3.0* (*N* = 10)	15.0 (*N* = 1)	0.00 ± 0.00 (*N* = 5)	1.30 ± 0.95 (*N* = 10)	3 (*N* = 1)

IOP: intraocular pressure; PI: peripheral iridotomy; PACS: primary angle closure suspect; CPAC: chronic primary angle closure; CPACG: chronic primary angle closure glaucoma.

^‡^Before any surgical treatment.

*Significantly different from baseline using paired *t*-test.
